# Supplementary material for: Improved Phenolic Profile, Sensory Acceptability, and Storage Stability of Strawberry Decoction Beverages Added with Blueberry Decoctions
Source: Molecules. 2023 Mar 9;28(6):2496. doi: 10.3390/molecules28062496 (PMC10051725; doi:10.3390/molecules28062496)
Supplement: Supplementary file 1 [file molecules-28-02496-s001.zip › molecules-2242901-supplementary.pdf]

SUPPLEMENTARY MATERIAL

Improved phenolic profile, sensory acceptability, and storage stability of strawberry decoction beverage added with blue-berry decoction

Ana M. Sotelo-González[1], Iza F. Pérez-Ramírez[1*], Julissa H. Soto-Infante[1], Haiku D. J. Gómez-Velázquez[2], Ma. Estela Vázquez-Barrios[1], Alexandro Escobar-Ortíz[1], Rosalía Reynoso-Camacho[1*]

[1]Chemistry School, Universidad Autónoma de Querétaro, Querétaro 76010, Querétaro, México.

[2]Institute of Neurobiology, Universidad Nacional Autónoma de México campus Juriquilla, Querétaro 76230, Querétaro, México.

[*]Correspondence: I.F.P.R.: iza.perez@uaq.mx; R.R.C.: rrcamachomx@yahoo.com.mx

Table S1. Acceptance index of the berry-based beverages by an untrained panel.

	Strawberry beverage					*Blueberry beverage*					*Strawberry-Blueberry beverage*			
**Scale**	*Consumers*	*Score*	*Average*	*AI*	*Scale*	*Consumers*	*Score*	*Average*	*AI*	*Scale*	*Consumers*	*Score*	*Average*	*AI*
1	0	0	0	0	1	0	0	0	0	1	0	0	0	0
2	0	0	0	0	2	0	0	0	0	2	0	0	0	0
3	0	0	0	0	3	0	0	0	0	3	0	0	0	0
4	5	20	0.4	4.44	4	2	8	0.16	1.78	4	1	4	0.08	0.89
5	3	15	0.3	3.33	5	5	25	0.5	5.56	5	2	10	0.2	2.22
6	7	42	0.84	9.33	6	4	24	0.48	5.33	6	6	36	0.72	8.00
7	11	77	1.54	17.11	7	23	161	3.22	35.78	7	21	147	2.94	32.67
8	12	96	1.92	21.33	8	11	88	1.76	19.56	8	11	88	1.76	19.56
9	12	108	2.16	24	9	5	45	0.9	10.00	9	9	81	1.62	18.00
Total	**50**	**358**	**7.16**	**79.56**	**Total**	**50**	**351**	**7.02**	**78.00**	**Total**	**50**	**366**	**7.32**	**81.33**

AI: acceptance index.

Table S2. Physicochemical parameters of the berry-based beverages stored at 4 °C for 90 days and 25 °C and 34 °C for 12 days.

Beverage	pH		°Brix		Titratable acidity*	
	Day 0	Day 90	Day 0	Day 90	Day 0	Day 90
			**4 ºC**			
Strawberry	3.51±0.07[a]	3.50±0.06[a]	4.08±0.07[a]	4.07±0.08[a]	0.36±0.02[a]	0.38±0.05[a]
Blueberry	3.57±0.10[a]	3.55±0.10[a]	3.40±0.19[a]	3.80±0.21[a]	0.18±0.01[a]	0.18±0.01[a]
Strawberry-Blueberry	3.44±0.03[a]	3.40±0.03[a]	3.63±0.29[a]	3.40±0.27[a]	0.27±0.02[a]	0.28±0.01[a]
			**25 °C**			
Strawberry	3.51±0.07[a]	3.05±0.03[b]	4.08±0.07[a]	3.71±0.25[b]	0.36±0.02[a]	0.36±0.00[a]
Blueberry	3.57±0.10[a]	3.10±0.02[b]	3.40±0.19[a]	2.74±0.07[b]	0.18±0.01[a]	0.19±0.00[a]
Strawberry-Blueberry	3.44±0.03[a]	3.01±0.08[b]	3.63±0.29[a]	3.17±0.00[b]	0.27±0.02[a]	0.28±0.02[a]
			**34 °C**			
Strawberry	3.51±0.07[a]	3.08±0.03[b]	4.08±0.07[a]	3.49±0.04[b]	0.36±0.02[a]	0.38±0.01[a]
Blueberry	3.57±0.10[a]	3.15±0.05[b]	3.40±0.19[a]	2.80±0.00[b]	0.18±0.01[a]	0.19±0.01[a]
Strawberry-Blueberry	3.44±0.03[a]	3.12±0.02[b]	3.63±0.29[a]	3.20±0.03[b]	0.27±0.02[b]	0.31±0.01[a]

Data are showed as mean ± standard deviation of three replicates. Different letters in each parameter in the same beverage indicate significant (p<0.05) differences. *g eq. citric acid/100 mL.

Table S3. Validation parameters of the standards used for the quantification of polyphenols by UPLC-ESI-QTOF MS^E

Standard	Regression model	Model	R2 coefficient	LOD (ng/mL)	LOQ (ng/mL)	Quantified compounds
Delphinidin chloride	y = 4549.10 +1794.86	Linear fit	0.9937	0.02	0.06	Anthocyanins
(-)-Epicatechin	y = 363.12 + 119.11	Linear fit	0.9899	0.01	0.04	(-)Epi-catechin
Procyanidin dimer B2	y = 322.33 + 129.53	Linear fit	0.9976	0.03	0.06	Procyanidin dimer B2
Quercetin	y = 561.11 + 179.17	Linear fit	0.9977	0.02	0.05	Flavonols
p-Coumaric acid	y = 3399.8 - 1116.21	Linear fit	0.9982	0.02	0.05	*p*-Coumaric acid derivatives
Ellagic acid	y = 2792.18 – 968.77	Linear fit	0.9991	0.01	0.03	Ellagic acid and ellagitannins

Calibration curves were constructed from six different concentrations for each standard and were analyzed by triplicate. LOD: limit of detection; LOQ: limit of quantification.

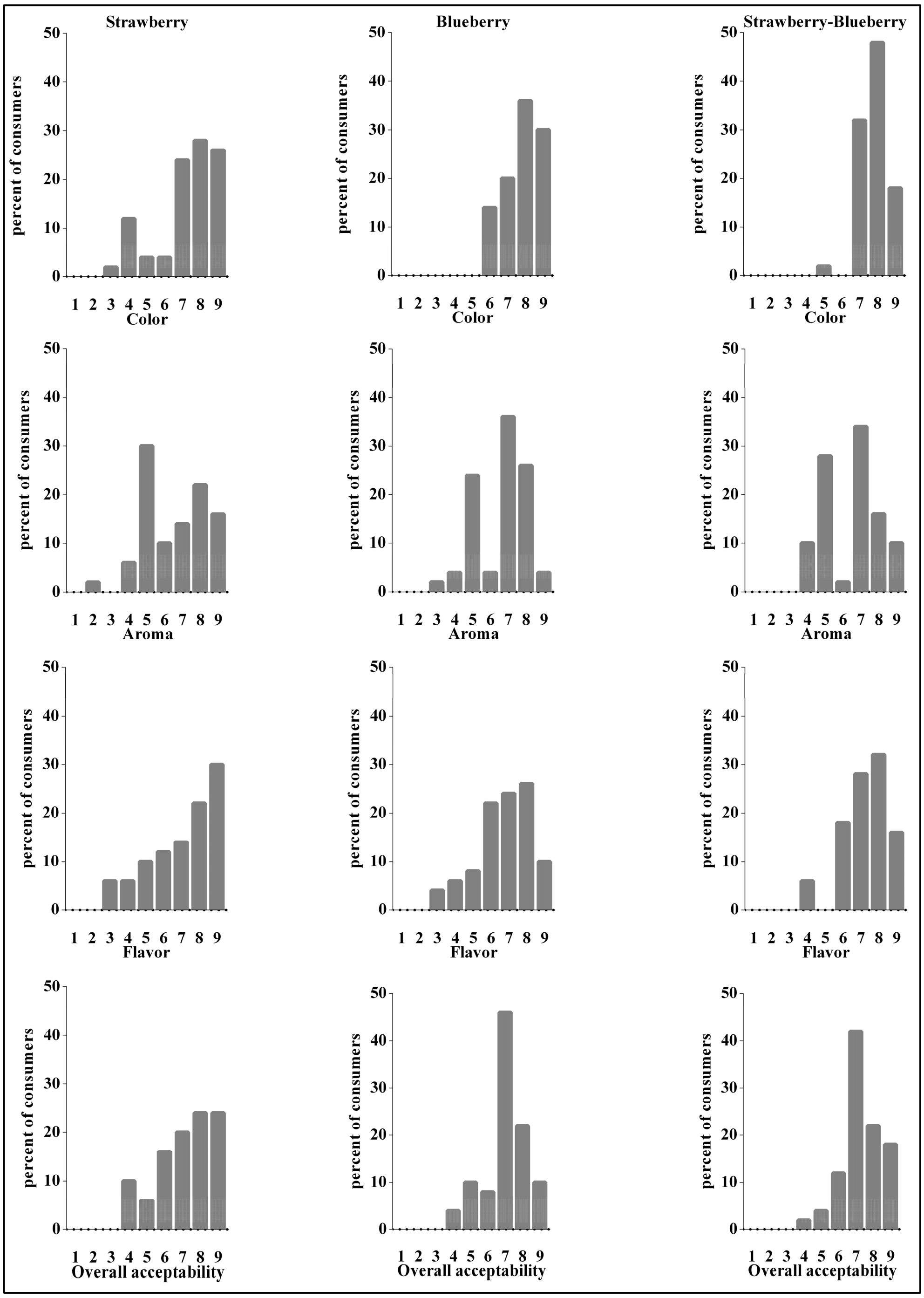

Figure S1. Category distribution of the 9-point hedonic scale of the berry-based beverages.
